# Valid 3D surface superimposition references to assess facial changes during growth

**DOI:** 10.1038/s41598-021-95942-3

**Published:** 2021-08-12

**Authors:** Simeon T. Häner, Georgios Kanavakis, François Matthey, Nikolaos Gkantidis

**Affiliations:** 1 Department of Orthodontics and Dentofacial Orthopedics, University of Bern, Freiburgstrasse 7, 3010 Bern, Switzerland; 2 Department of Pediatric Oral Health and Orthodontics, UZB – University School of Dental Medicine, University of Basel, 4058 Basel, Switzerland; 3 Department of Orthodontics, Tufts University School of Dental Medicine, Boston, MA 02111 USA; 4 Private Practice, Neuchâtel, Switzerland

**Keywords:** Outcomes research, Anatomy

## Abstract

Currently, the primary techniques applied for the assessment of facial changes over time utilize 2D images. However, this approach has important limitations related to the dimensional reduction and the accuracy of the used data. 3D facial photography has been recently introduced as a risk-free alternative that overcomes these limitations. However, the proper reference areas that should be used to superimpose serial 3D facial images of growing individuals are not yet known. Here, we tested various 3D facial photo superimposition reference areas and compared their outcomes to those of a standard anterior cranial base superimposition technique. We found that a small rectangular area on the forehead plus an area including the middle part of the nose and the lower wall of the orbital foramen provided comparable results to the standard technique and showed adequate reproducibility. Other reference areas that have been used so far in the literature were less reliable. Within the limitations of the study, a valid superimposition reference area for serial 3D facial images of growing individuals is suggested. The method has potential to greatly expand the possibilities of this highly informative, risk free, and easily obtained 3D tool for the assessment of facial changes in growing individuals.

## Introduction

Facial appearance and facial expressions have a great impact on most daily human interactions. The attractiveness of the face in particular affects various aspects of life, including social, romantic and professional decisions^1^. Therefore it is not surprising that facial appearance is considered an important factor for patient satisfaction in health care disciplines focused on the craniofacial area^2^.

The above considerations indicate the utmost importance of proper facial soft tissue imaging. Despite their limitations regarding dimensional reduction and loss of structural information, conventional 2D (two-dimensional) imaging modalities, such as 2D cephalometry and facial photography, are still the most commonly used methods for assessing facial soft tissues and evaluating treatment effects on facial appearance^3^. However, with the advent of recent technological advancements, 3D (three-dimensional) methods are rapidly gaining attention offering a number of advantages, such as the reduced amount of distortion, the fact that image correctness is not dependent on head position, but most importantly the ability to evaluate the face in all three dimensions^4,5^. Three-dimensional facial soft tissue data can nowadays be acquired easily with several techniques, such as 3D facial stereophotogrammetry. Most are non-invasive, risk-free, accurate and easy to obtain, and are thus likely to become widely used in everyday practice^6–8^. This will greatly expand the imaging possibilities of clinicians and researchers in various fields including plastic and maxillofacial surgery, and orthodontics. Currently, disadvantages of 3D imaging derive from the fact that usually the equipment is more expensive, difficult to access, and sometimes special training, as well as software, is required to acquire and process the images. Furthermore, due to the relatively low penetration in the market, sometimes hardware and software support is limited.

In order to thoroughly study treatment outcomes or physiological facial changes over time, a superimposition of a series of patient images is needed. Three dimensional superimposition techniques utilize the whole amount of 3D information for a detailed assessment and visualization of facial changes. Thus, they consist a valuable tool for treatment planning and outcome assessment. However, superimposition of serial facial images should ideally be performed on a morphologically stable reference area. Then, changes in the neighboring structures can be precisely visualized and measured through color coded distance maps^4,5,9–11^.

Several studies in the literature have reported techniques to evaluate serial 3D facial photos/surfaces^12–18^. Most available studies used the forehead and parts of the glabella or nasal structures as reference areas to superimpose images of non-growing patients and/or within a short examination period of 6 months or less^12–16,18^. Other researchers used a surface-based best-fit registration approach using the entire face, but also within a very short time frame, without expecting any morphological change in-between^17^. Therefore, the evidence supporting the adequacy of such methods is limited, especially in growing patients and for medium- to long-term assessments, where facial changes are expected to occur even without any intervention.

Based on the current evidence, it would not be overstated to say that so far none of the reported 3D facial image superimposition techniques has been thoroughly tested for its validity, its adequacy in evaluating medium- to long-term changes or its applicability in growing patients. Furthermore, there is no study comparing two facial surface superimposition methods to each other or to another method. Therefore, the present investigation assessed various 3D facial superimposition reference areas in growing patients, using CBCT derived data, and compared them to a voxel-based anterior cranial base (ACB) superimposition, which is considered the gold standard approach^4,9,19^.

## Materials and methods

### Study design and ethical approval

This project is a prospective methodological study based on retrospectively obtained radiographic data and has been approved by the Swiss Ethics Committees (Protocol No. 2018-01670). The methods were carried out in accordance to the relevant guidelines and regulations and participants signed an informed consent prior to the use of their data in the study.

### Sample

To collect the study sample the following inclusion criteria were applied on a preexisting archive of a single orthodontic clinic in Switzerland, considering consecutively obtained data between 2008 and 2018: (a) age between 11 and 12.5 years at the first CBCT scan (T0), (b) time span between T0 and T1 (second CBCT scan) from 1 to 3 years, and (c) scans with teeth in slight contact at maximum intercuspation and the facial soft-tissues at rest. The exclusion criteria were: (a) scans with any facial expression or muscle tension at any time point, (b) scans with any appliance in the mouth during image acquisition, (c) participants wearing appliances that contact the facial soft-tissues, such as Dellaire mask or chin cup, (d) scans of insufficient diagnostic quality (increased noise, artifacts, or distortion) regarding craniofacial morphology, and (e) craniofacial syndromes, congenital malformations, severe facial asymmetries, or systemic diseases that might affect craniofacial morphology. Two operators inspected all criteria independently (S.H. and N.G) and any disagreements were resolved by consensus.

Eighteen pre-existing pairs of serial craniofacial CBCT images fulfilled the inclusion criteria and comprised the study sample. Sample selection was based on availability. Since there is no comparable study available, sample size calculation was not possible. This sample size was considered adequate based on studies testing analogous hypotheses^5,20^. All subjects were growing orthodontic patients (average age at T0: 11.7 ± 0.6 years old; sex: 9 males, 9 females) and the average time span between two corresponding images of the same individual was 1.7 ± 0.5 years. All sagittal and vertical maxillofacial growth patterns were considered for inclusion. Detailed sample characteristics are provided in Table 1.

**Table 1:** Detailed sample characteristics.

Sagittal facial type	Class I (%)	Class II (%)	Class III (%)	Total
Dental class^1^	7 (38.9)	10 (55.6)	1 (5.6)	18
Skeletal class^2^	5 (27.8)	8 (44.4)	5 (27.8)	18
	**Hypodivergent (%)**	**Normodivergent (%)**	**Hyperdivergent (%)**	
**Vertical facial type**^**3**^	0 (0)	12 (66.7)	6 (33.3)	18
	**Narrow (%)**	**Normal (%)**	**Wide (%)**	
**Transversal facial type**^**4**^	0 (0)	17 (94.4)	1 (5.6)	18

^1^Deviation less than ¼ of a molar cusp was considered Class I.

^2^Class I: 1° ≤ ANB ≤ 6°, Class II: ANB > 6°, Class III: ANB < 1°.

^3^Hypodivergent: MP-PP < 20°, Normodivergent: 20° ≤ MP-PP ≤ 30°, Hyperdivergent: MP-PP > 30°.

^4^Narrow: Zy–Zy < 118 mm, Normal: 118 ≤ Zy–Zy ≤ 131 mm, Wide: Zy–Zy > 131 mm; Zy–Zy: soft-tissue interzygomatic distance, defined by Farkas et al. (1992)^21^.

The CBCTs were acquired by the same X-ray machine (KaVo 3D eXam, Hatfield, PA 19440, USA), when it was considered to offer valuable information that could affect clinical decisions. The ALADA (As Low as Diagnostically Acceptable) principle was followed. All CBCTs had a voxel size of 0.4 mm and were performed with a 3.5 mA tube current, 120 kV tube voltage and a field of view of 170 mm height by 232 mm diameter. The scan time was 8.9 s with an exposure time of 3.7 s. All data were stored in DICOM files.

### Voxel-based ACB superimposition

Voxel-based registration of serial CBCT images was performed through Dolphin 3D software^©^ (Version 2.1.6079.17633, Dolphin Imaging and Management Solutions Chatsworth, CA 91311 U.S.A). The pairs of CBCTs (T0 and T1) were superimposed on the anterior cranial base (ACB) using a previously validated voxel-based best fit approach^4,19,22^. Since the anterior cranial base is morphologically stable already at an early age^23,24^, it is located centrally in the craniofacial complex, and it has a steady relation to the natural head position^25^, superimposition in this area is considered a gold standard for the assessment of maxillofacial structures^5,9^. The selection of the reference area was performed on the CBCT T1, in a multiplanar view and consisted of a rectangular box of voxels, which included the anterior cranial base^4,19^. CBCT T0 was then relocated to the position of the cranial base of CBCT T1, following a best fit registration of the two models on the selected voxels.

Soft tissue surfaces were segmented from the superimposed CBCT models (T0 und T1). The surface segmentation was performed manually by one operator (S.H.) by varying the threshold until the facial surface was smooth and with the least amount of noise or distortion^19^. Surface data were exported in an STL format.

### Reference area selection

**Figure 1:**
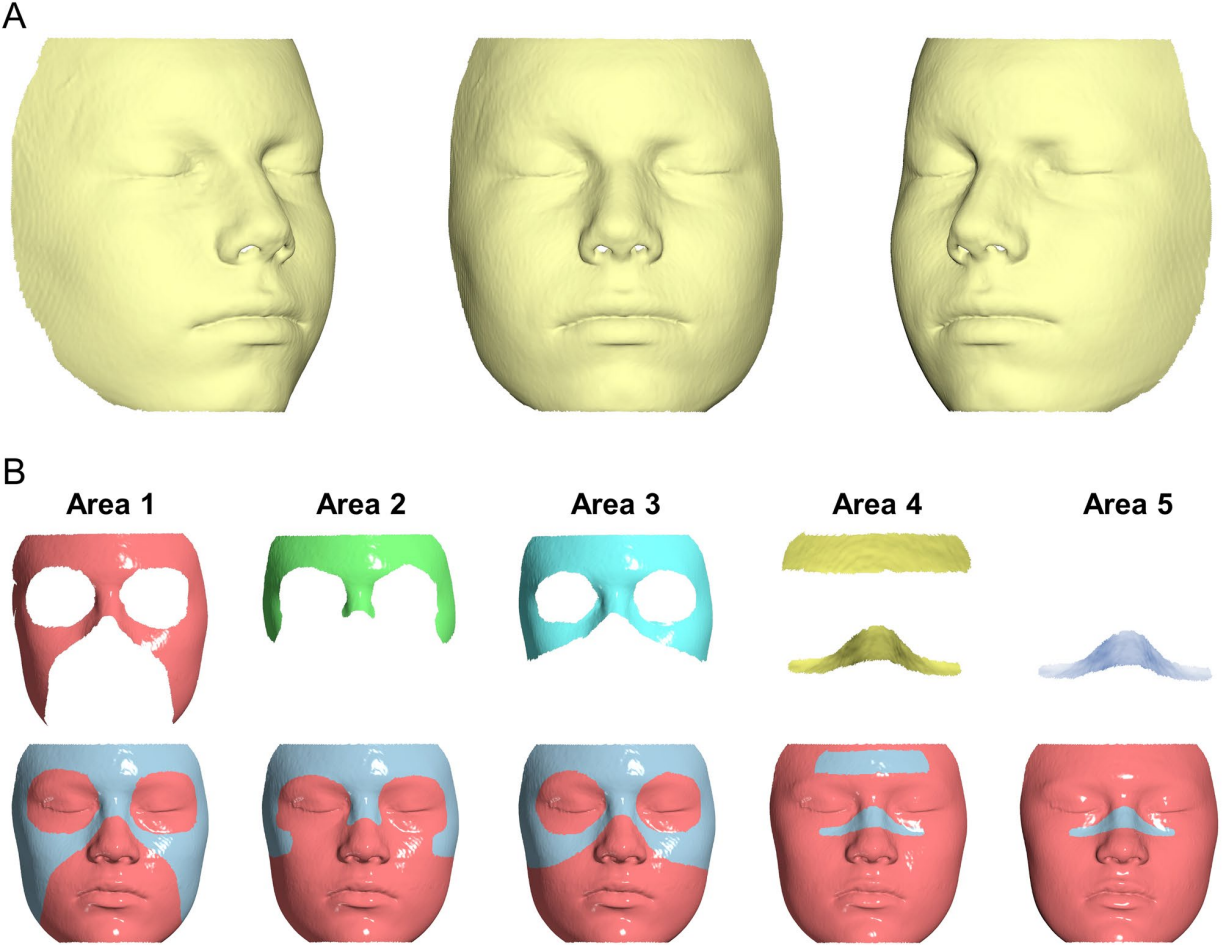
(**A**) Example of a cropped CBCT derived facial surface model that was used in the study. (**B**) Facial surface superimposition reference areas tested in the study. At the last row, the light blue color indicates the selected reference area. All images were generated using Viewbox 4 software (version 4.1.0.1 BETA, http://www.dhal.com/viewboxindex.htm).

For further assessment, all facial surface models were imported in *Viewbox 4* Software (version 4.1.0.1 BETA 64, dHAL software, Kifisia, Greece), which has been shown previously to properly process surface data^4,11,19,26–28^. The T1 surface model was cropped to include only the facial areas of interest (Fig. 1A). The cropped surface model was then duplicated 4 times for selecting on each subsequent model, one of the five following superimposition reference areas: (1) Whole facial surface excluding the eyes, the mouth and the tip of the nose, (2) Forehead glabella and base of the nose, (3) Upper half of the face excluding the eyes and the tip of the nose, (4) A small rectangular area on the forehead and an area including the middle part of the nose and the lower wall of the orbital foramen, (5) Same as area 4, but without the area on the forehead (Fig. 1B). These references areas were determined according to previous literature^15,29,30^, as well as to the determination of the most stable facial soft tissue structures relevant to the anterior cranial base, following the ACB voxel-based superimpositions of the present study. The reference area selection on each T1 surface model was performed manually by one operator (S.H.), based on the corresponding anatomical definitions.

### Facial surface superimposition

Afterwards, each T1 facial surface model was superimposed to the T0 model on the reference area selected each time, using a best fit ICP (Iterative Closest Point) approach^31^, with the following settings: 100% estimated overlap of meshes, matching point to plane, exact nearest neighbor search, 100% point sampling, exclude overhangs, 50 iterations. The algorithm was repeatedly applied (usually 4–5 times) until a minimum distance between corresponding T0-T1 surfaces was reached. The T0 model was always stable and the T1 model was relocated during the superimposition process.

### Data analysis

The outcomes of the five different surface superimposition techniques regarding facial changes between T0 and T1 were visually assessed by the first and the last author, independently, following previous calibration. This was performed through the comparison of the corresponding colour coded distance maps to those of the ACB voxel-based anterior cranial base superimposition, which was the gold standard for the study. Based on the outcomes of this assessment, the gold standard method and three surface superimposition reference areas, which were considered comparable to the gold standard, were also assessed quantitatively as described below. The mean absolute distances (MAD) between the superimposed T0–T1 models were measured at the following seven areas, distributed over the whole facial soft tissue surface (100 vertices each): Soft tissue N-point, soft tissue A-point, soft tissue pogonion, soft tissue zygomatic arch right and left, and soft tissue gonial angle right and left^19^. For each specific subject, these areas were selected once at the T0 model and were then duplicated for consistency reasons.

Having the T0 models always at the same position, color coded distance maps were also created to directly visualize the differences of the anterior cranial base registered T1 models and those registered on different facial surface areas. These were first qualitatively assessed and then, MADs of the three most comparable facial surface superimposition techniques to the ACB voxel-based outcome were calculated in the same manner, as described above.

To further assess the T0-T1 surface congruence of the best performing reference area, the MAD of the surface-based superimposed serial models at this area was measured.

### Intra-operator reproducibility of the surface-based superimposition

The voxel-based superimposition method used in this study has been previously validated thoroughly^4,19^. To assess the intra-operator reproducibility of the surface-based superimposition, facial surface models from ten randomly selected CBCTs were segmented and superimposed a second time, as described above. MADs between the T0 and T1 models at the seven previously described areas were calculated for the different superimposition techniques. Intra-operator reproducibility was assessed by calculating the differences between the MAD values obtained after 1st and the 2nd superimposition.

### Statistics

Statistical analysis was carried out with SPSS Software (IBM SPSS Statistics for Windows, Version 25.0. Armonk, NY: IBM Corp.). Raw data were tested for normality of distribution through the Kolmogorov–Smirnov and Shapiro–Wilk tests.

Evidence of non-normality was present, and thus, non-parametric statistics were applied.

Differences among more than two measured variables were evaluated using Kruskal–Wallis test. When significant differences were detected, pair-wise a-posteriori comparisons were performed through Dunn–Bonferroni post hoc test, which accounts for the possibility of false-positive results in multiple comparison tests.

In all cases, a two-sided significance test was carried out at an alpha level of 0.05.

## Results

**Figure 2:**
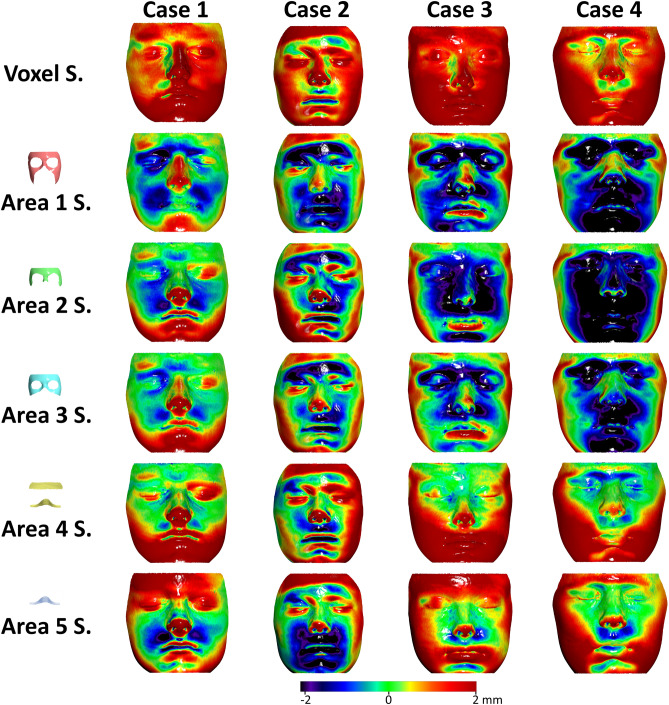
Colour coded distance maps showing T0–T1 facial surface changes of four representative cases, as detected by anterior cranial base voxel-based or five different facial surface-based superimpositions. The T0 facial surface model was used as a reference. S.: Superimposition. All images were generated using Viewbox 4 software (version 4.1.0.1 BETA, http://www.dhal.com/viewboxindex.htm).

**Figure 3:**
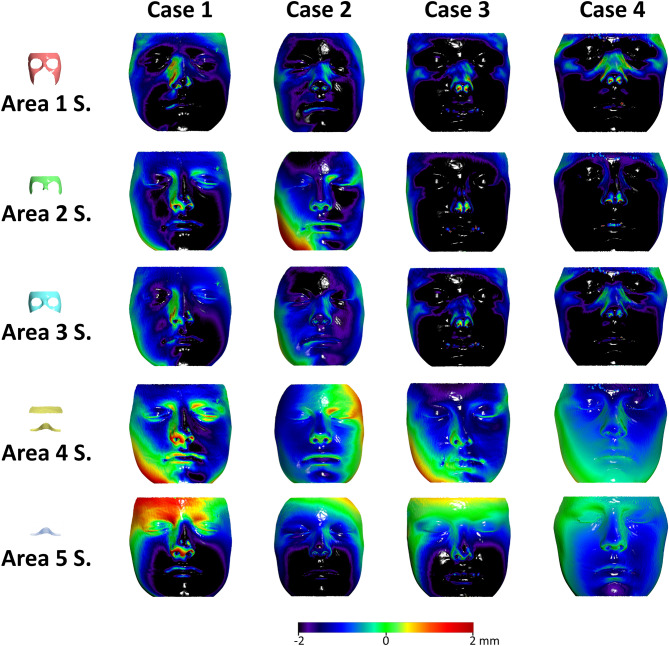
Colour coded distance maps showing the differences in the outcomes (T1 models) of the five different facial surface-based superimpositions from the anterior cranial base voxel-based superimposition, on four representative cases. The voxel-based superimposition T1 surface model was used as a reference. S.: Superimposition. All images were generated using Viewbox 4 software (version 4.1.0.1 BETA, http://www.dhal.com/viewboxindex.htm).

According to two independent assessors, the qualitative assessment of the colour coded distance maps showing the T0–T1 changes detected by the ACB voxel-based and the five surface-based superimposition techniques showed a clear inconsistency between Area 1 and Area 2 surface-based techniques and the voxel-based technique. There was no specific pattern detected in these inconsistencies. Four cases are shown as examples in Fig. 2 and the rest of the sample is presented in Supplementary Figs. 1–4. Similar findings were evident through the visual assessment of the colour coded distance maps showing the differences in the outcomes (T1 models) of the five different facial surface-based superimpositions from the ACB voxel-based superimposition (Fig. 3 and Supplementary Figs. 5–8). Based on the above assessments it was a joint decision of the authors to not further analyse Area 1 and Area 2 surface-based superimposition techniques.

**Figure 4:**
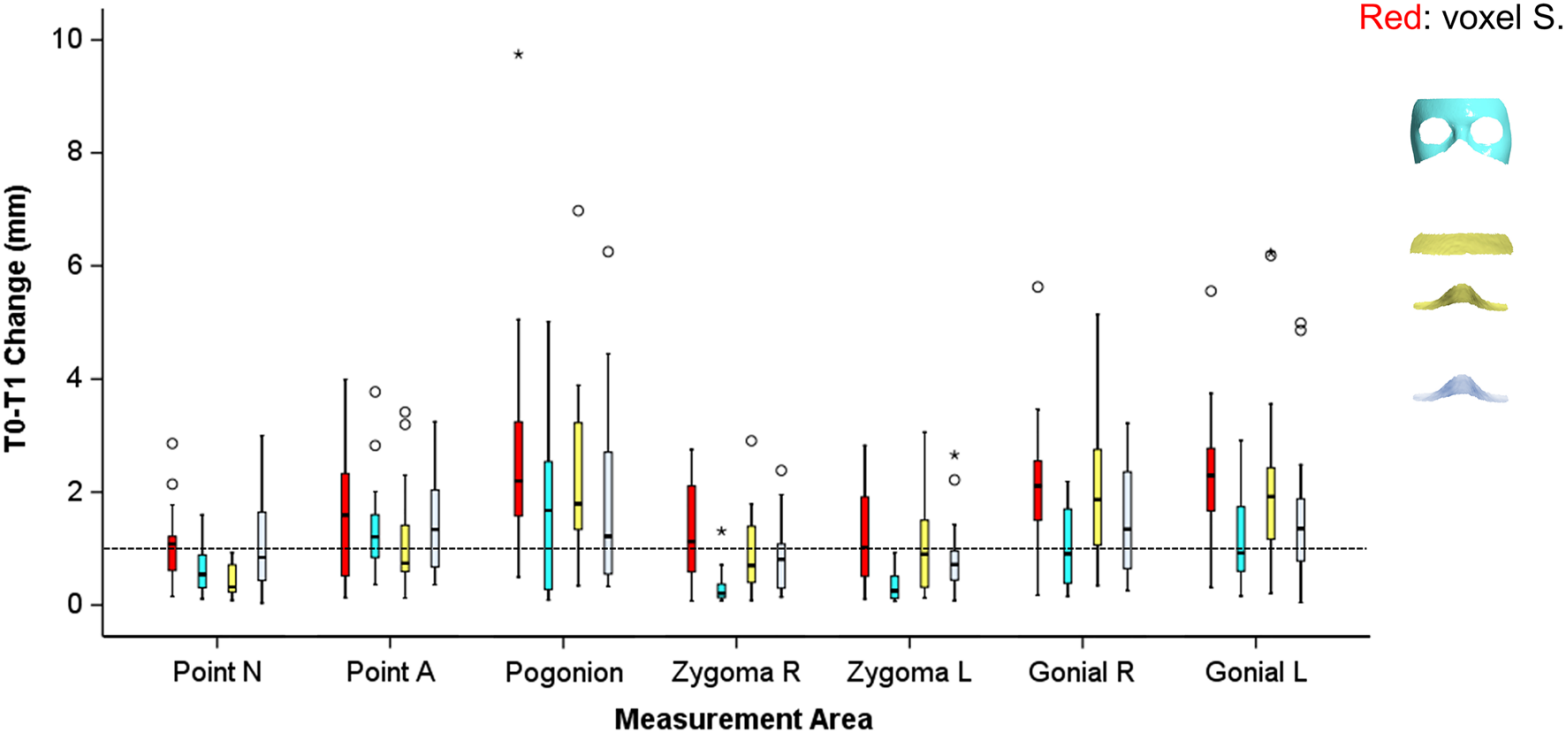
Box plots showing in the y-axis the T0-T1 changes detected through the anterior cranial base voxel-based superimposition and the three surface-based superimpositions. The upper limit of the black line represents the maximum value, the lower limit the minimum value, the box the interquartile range, and the horizontal black line the median value. The dashed horizontal line indicates changes of 1 mm. Outliers are shown as black circles or asterisks, in more extreme cases, with a step of 1.5 × IQR (interquartile range).

**Figure 5:**
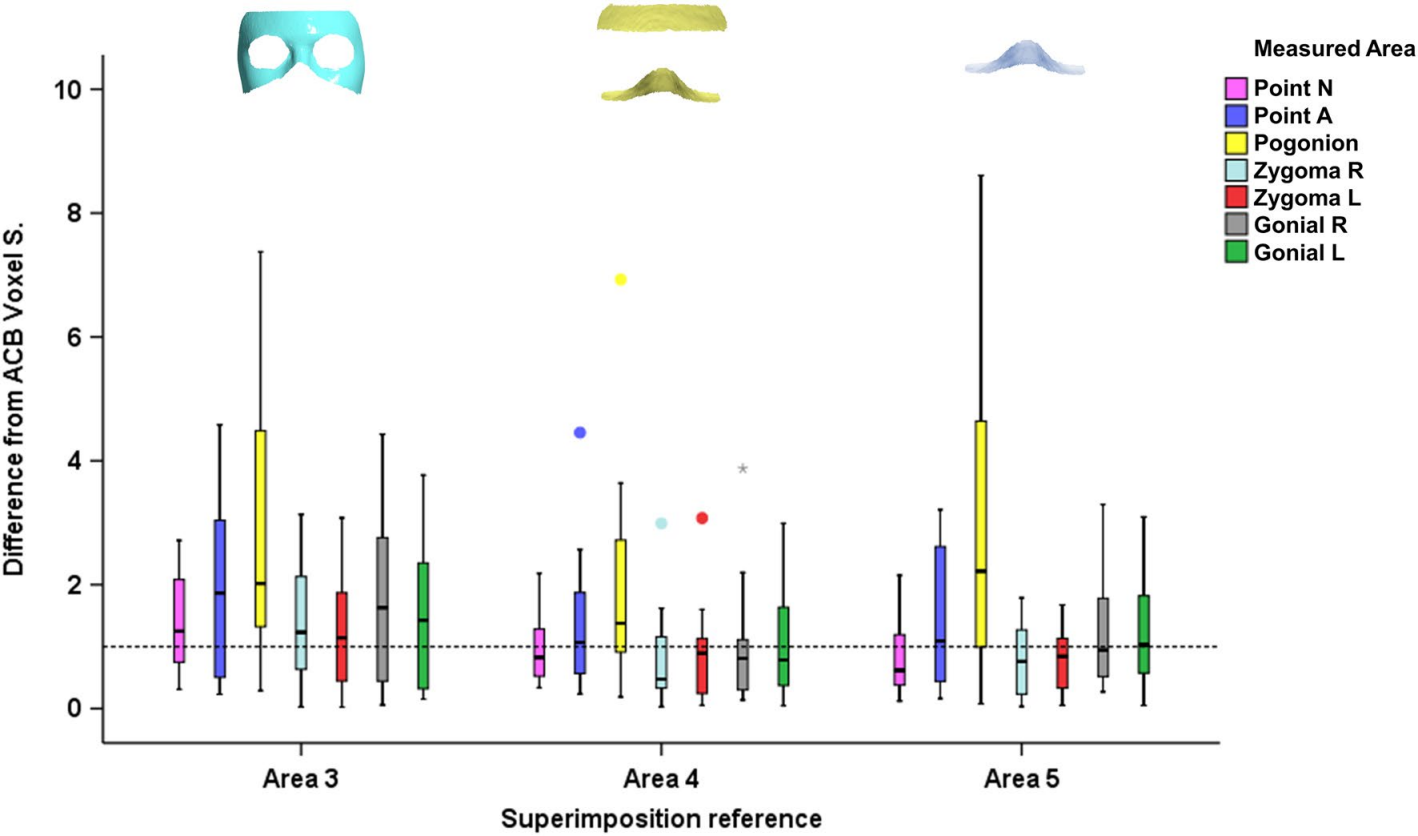
Box plots showing in the y-axis the difference of the ACB voxel from the surface based superimposition techniques. The upper limit of the black line represents the maximum value, the lower limit the minimum value, the box the interquartile range, and the horizontal black line the median value. The dashed horizontal line indicates difference of 1 mm. Zero value indicates perfect agreement with the gold standard. Outliers are shown as circles or asterisks, in more extreme cases, with a step of 1.5 × IQR (interquartile range). The vertical length of each plot indicates the variation of the differences.

There were significant differences in the overall T0–T1 changes detected at the seven measurement areas by the ACB voxel-based superimposition and the three quantitatively tested surface-based superimposition techniques (Fig. 4; ACB median: 1.65, range: 0.07, 9.75 mm; Area 3 median: 0.69, range: 0.07, 5.01 mm; Area 4 median: 1.10, range: 0.08, 6.98 mm; Area 5 median: 0.98, range: 0.04, 6.25 mm; Kruskal Wallis test: p < 0.001). Significant differences in the T0–T1 changes were also detected among techniques when testing individual measurement areas (Fig. 4; Kruskal Wallis test; Point N: p = 0.008, Point A: p = 0.671; Pogonion: p = 0.188; Zygoma R: p = 0.001; Zygoma L: p = 0.000; Gonial R: p = 0.015; Gonial L: p = 0.018). Areas 4 and 5 clearly showed higher agreement to the ACB voxel-based technique, than Area 3, with Area 4 performing slightly better. This was also evident when directly comparing the ACB voxel to the surface-based superimposition outcomes through differences in the corresponding T1 models (Area 3 median: 1.37, range: 0.02, 7.38 mm; Area 4 median: 0.88, range: 0.03, 6.93 mm; Area 5 median: 0.97, range: 0.03, 8.61 mm; Kruskal Wallis test: p = 0.001). In this case, when the individual measurement areas were tested separately, most differences did not reach the statistical significance level (Fig. 5, Table 2; Kruskal Wallis test; Point N: p = 0.046, Point A: p = 0.546; Pogonion: p = 0.327; Zygoma R: p = 0.100; Zygoma L: p = 0.196; Gonial R: p = 0.375; Gonial L: p = 0.551).

**Table 2:** Sum of differences (mm) of each surface superimposition outcome from the ACB voxel-based superimposition outcome in each of the seven areas measured (n = 18).

	N	A	Pog	ZygR	ZygL	GoR	GoL	Total
Area 3	25.57	34.15	50.09	23.71	22.25	28.45	27.08	211.29
Area 4	16.98	24.86	35.27	13.80	15.74	17.17	19.47	143.28
Area 5	15.28	25.95	53.66	14.38	14.00	22.60	21.39	167.26

The colour coded distance maps showing the T0–T1 changes detected by the ACB superimposition, indicated that in most cases the entire facial surface, moved slightly forward (Fig. 2 and Supplementary Figs. 1–4). For this reason, all facial surface superimposition techniques showed in most cases a more retruded position of the T1 surface model compared to that shown by the ACB technique (Fig. 3 and Supplementary Figs. 5–8). This was also evident for Area 4 in 12 of the 18 cases tested. Apart from this, the difference of the best performing Area 4 to the ACB voxel-based technique did not show any consistent spatial pattern. On the contrary, differences between Areas 4 and 5 were mostly located at the lower part of the face, with Area 5 showing a clockwise rotation of the face around the transversal axis, compared to the ACB voxel-based technique. Area 3 showed consistently higher deviations from the ACB voxel-based technique in all areas (Figs. 3,4,5, Table 2).

**Figure 6:**
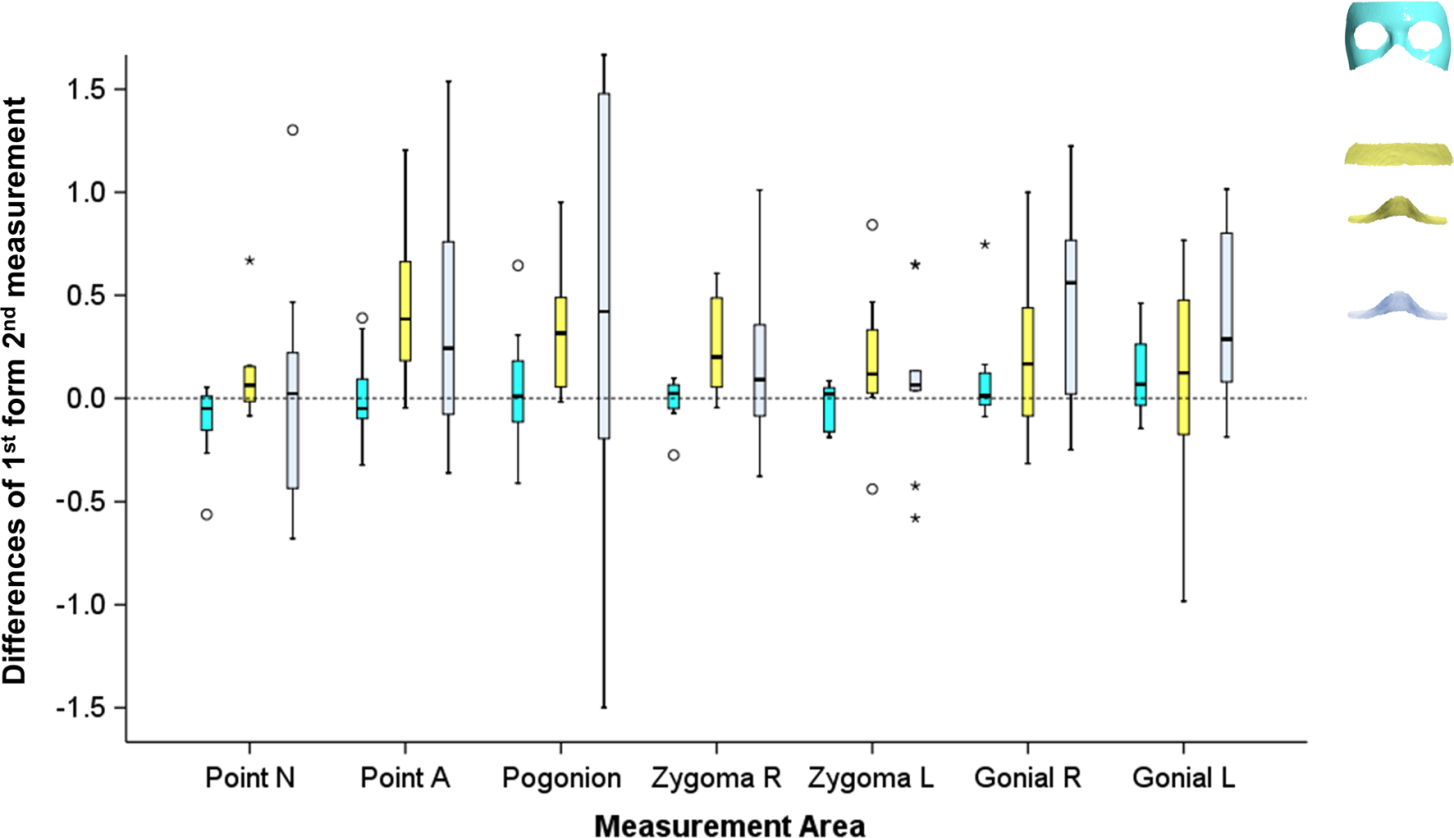
Box plots showing in the y-axis the differences between repeated facial surface model segmentation and superimposition on three references areas. The dashed horizontal line indicates zero, and thus, perfect reproducibility. The upper limit of the black line represents the maximum value, the lower limit the minimum value, the box the interquartile range, and the horizontal black line the median value. Outliers are shown as black circles or asterisks, in more extreme cases, with a step of 1.5 × IQR (interquartile range). The vertical length of each plot indicates imprecision.

When T0–T1 surface models were superimposed on Area 4, the median distance of the models on this area was 0.33 mm (range: 0.021, 0.70 mm), indicating adequate congruence of the serial facial surface models on this area.

The reproducibility of the tested surface-based superimposition methods, including the segmentation error, was in most cases within 0.5 mm and it was considered acceptable (Fig. 6). However, there were significant differences between the techniques (Kruskal Wallis test, p < 0.001) with Area 3 (median: 0.01 mm; range: − 0.56, 0.75 mm) being more precise than Areas 4 (median: 0.18 mm; range: − 0.98, 1.20 mm) and 5 (median: 0.10 mm; range: − 1.50, 2.00 mm) (Dunn–Bonferroni post hoc test, p < 0.003). Areas 4 and 5 showed similar median reproducibility levels (p > 0.05), but Area 5 showed higher inconsistency in individual cases (Fig. 6).

## Discussion

The face is a key component of an individual’s attractiveness and largely affects human interactions and relationships^1,32–34^. Facial shape, symmetry and averageness are few of the factors that are related to facial attractiveness^1,35^ and guide patients’ and clinicians’ opinions about physical appearance. Enhancing facial attractiveness is an important motivating factor for patients to seek treatment^36,37^, thus making the proper assessment of facial soft tissues one of the most important parts of a thorough clinical examination.

Three dimensional surface imaging techniques have greatly expanded the possibilities for accurate, easily acquired, and risk free 3D facial imaging. The superimposition of serial 3D images consists a valuable tool for the evaluation of individual facial changes over time^4,5,9,38^. Among others, this facilitates treatment decision-making and prediction of outcomes for ongoing or future interventions. The present study investigated, for first time, various 3D facial surface superimposition areas in growing individuals and compared their applicability to those of a standard 3D anterior cranial base superimposition technique^5,9,23,27^, which has been previously validated^4,19,22^.

A small rectangular area on the forehead plus an area including the middle part of the nose and the lower wall of the orbital foramen (Area 4) was found to show morphological stability, adequate reproducibility, and comparable results to the golden standard technique. Though close to the facial center, this area is located towards the upper third of the face, allowing for proper assessment of the middle and lower parts of the face which are primarily affected by treatment and growth^39,40^, and show the highest variation in the human face^41^. This superimposition reference is similar to that recommended by the 3dMD camera manufacturer, consisting of an analogous forehead area and the nasal bridge^12,42^. Nevertheless, we do not consider the latter superior to the area suggested here, because the nasal bridge often presents a different growth pattern than the forehead^43,44^, which may thus confound the superimposition outcomes. Furthermore, the larger area that is suggested here has been verified according to the standard ACB superimposition and is also more robust to artifacts^26^. Finally, increased artifacts might be expected on the nasal bridge area in certain individuals due to facial hair.

Area 3, which was the largest area tested, showed better reproducibility than Areas 4 and 5. Area 5, which was the smallest, also showed higher inconsistency in individual cases than Area 4. This was expected since larger surfaces that are used as superimposition references are more robust against artifacts and errors^26^. This fact, along with the results of the comparisons to ACB superimposition clearly favored Area 4 as the most appropriate reference area. It should be noted that reproducibility in this study involved superimposition reference area selection, measurement area selection, and segmentation error. According to previous studies, without the last three sources of error, the reproducibility of the surface superimposition methods would have been perfect^4,11,19,26–28,38^. When 3D photos are used instead of CBCT derived facial surface models, there is no segmentation error, though this is anyway of limited extent^19^. Thus, the detected reproducibility levels can be considered quite satisfactory.

Here, the CBCT derived facial surface models were used to assess the outcomes of 3D facial image superimposition techniques as related to the internal skeletal configuration. It has been shown that CBCT derived facial soft-tissue surface models are quite similar to those obtained by stereophotogrammetry^16,45,46^, allowing the assessment of changes in facial morphology in regards to standard reference structures, namely the anterior cranial base^4,5,9,19^. Thus, through the comparison with well-established anterior cranial base superimposition techniques^4,5,9,19^, the performance of the surface-based techniques was thoroughly assessed.

Previous studies have used the whole facial surface to superimpose serial facial images of the same individual^17,47,48^. This approach cannot be recommended, especially for growing individuals, since changes in different areas of the face are averaged following the application of a best-fit algorithm. To draw clinically relevant conclusions from a superimposition, this should be performed on relatively form-stable reference areas that also have a biological rationale^49^, as is true for the method suggested here^9^. Following the best possible matching of serial 3D images on these areas, changes that occurred over time in other areas can be assessed. Current facial growth and development concepts suggest that the middle and lower thirds of the face change considerably during growth^41^. Thus, if these parts of the face are included in the superimposition reference area, other parts of the face that were relatively unaltered will appear as being changed. On the other hand, changes in actually altered areas will appear reduced, following a best fit that also included unchanged areas. For these reasons, the superimposition methods that we used were primarily located at the middle and the upper parts of the face.

The present study tested a group of individuals in active growth, with a reasonable intermittent period of 1–3 years between the superimposed images. We considered this period clinically relevant for several disciplines, such as orthodontics and maxillofacial surgery. The findings of the present study are expected to also be applicable for shorter time periods and the suggested methods to present equivalent, if not better performance. However, further research is needed to confirm the applicability for longer time periods between consecutive images.

In line with previous research findings testing the application of surface-based superimposition techniques in neighboring areas^11,38^, the present study concluded that the selection of the reference area considerably affects the outcomes. We showed that even small differences in the superimposition reference areas may have considerable effects. Thus, careful selection of the used reference area should be emphasized. Additionally, it is questionable that the quite different areas that have been used so far in the literature, all perform as well as claimed.

So far, few previous studies have tested different landmark^15,17,29^ or surface-based^15,17,47,48^ superimposition techniques for serial 3D facial images of an individual. Most existing studies introduced a method as reliable, but they usually tested the reproducibility of a single method, they lacked a standard reference method for outcome comparisons, or only tested group mean differences and not the performance of the method in individual cases^12,15,17,29,47,48^. Furthermore, there is a high heterogeneity among the existing studies in the characteristics of the tested groups, the time span between serial images and the applied methods. In the existing literature, there was no study that tested more than one surface-based technique, though this approach has been shown to be superior to landmark-based techniques^5^.

An important strength of the present study is that a widely used and tested, standard superimposition technique in growing individuals has been used here as a reference for comparisons^5,9,19,23,27,41^. The anterior cranial base is a conserved modular structure^41^, located at the center of the head and it ceases growth early in development, at approximately 7.5 years of age^23,24^. Furthermore, it shows a constant relationship over time to natural head position^50,51^, which represents the primary way people present themselves during everyday life interactions. These features have designated the anterior cranial base area as the gold standard, both for growing and non-growing individuals^5,9,19,23,27,41^. For this reason, we validated various facial surface superimposition techniques based on the comparison of their outcomes to those of the anterior cranial base superimposition. We suggest this methodology as the most appropriate, evidence-based approach to investigate the performance of facial surface superimposition reference areas on different occasions.

There was no surface superimposition technique that showed identical outcomes to the anterior cranial base technique. This was expected and it should not be considered a limitation of the surface-based techniques. The facial surface of an individual might change due to muscle tension alterations or due mimicry even when considering consecutive image acquisition. Maal et al. 2011^52^ compared the variance of different regions of the facial soft tissue when repeating the same rest 3D facial photo and concluded that the forehead and the nasal area seem to show less variance. This supports the superiority of Area 4, as suggested here. Furthermore, even within a single day changes might be evident due to the variation in the hydration status of the soft-tissues. Weight fluctuations can also affect the morphology of the face, even in short term^53^. Additional to these factors, in growing individuals, changes in the skeletal and soft-tissue configurations also affect the facial surface.

Indeed, the colour coded distance maps showing the changes detected by the ACB superimposition, indicated that in most cases the entire facial surface, moved slightly forward (Fig. 2 and Supplementary Figs. 1–4). This might be attributed at a thickening of the hard and soft tissues between the ACB and the facial surface during the tested period. The opposite was true for Case 13 shown at Supplementary Fig. 7, where the depicted individual lost a lot of weight during the assessment period. The suggested Area 4 includes areas of thin soft-tissues over the underlying bone and minimal adiposity, and thus, it is less influenced by potential weight fluctuation between the two serial photos. This might be the reason why Area 3 consisting of a part of the upper cheek, which shows increased fat apposition, did not perform that well in the comparison to the ACB superimposition. Area 5 was not expected to be highly affected by weight fluctuations, but it showed significant rotations of the face compared to ACB outcomes. This is attributed to the small extent of this area which makes it vulnerable to local morphological changes over time^26,43,44^.

On the other hand, the anterior cranial base remains morphologically unaltered already after a young age^23,24^. A certain difference of the anterior cranial base superimposition to a facial surface superimposition might be expected, as described above. Nevertheless, such a comparison is still valuable, not only to compare the magnitude of differences detected by the different techniques, but also to verify that the direction of the changes is not significantly skewed due to rotations of the models related to local facial surface changes at the superimposition reference areas. The constant relation of the anterior cranial base to the natural head position over time^25,51^ verifies that such an occurrence could be adequately detected through the performed comparisons. Based upon the verified relationship between Area 4 and natural head position and on the fact that the facial surface is the one in direct contact to the human eye during interpersonal communication, it may be suggested that the outcomes of such a facial surface superimposition are clinically more representative of facial appearance than those of the ACB superimposition.

Another main strength of the facial surface superimposition over the ACB superimposition is the risk-free and rapid image acquisition. The ACB can only be captured through radiographs which require radiation exposure, and thus have associated risks. Furthermore, radiographs require more time, which introduces motion artifacts to the acquired image^54^. On the other hand, a limitation of the surface imaging could be that the surface might be disturbed in case of facial hair development over time.

### Limitations

A limitation of the present study is that it used CBCT derived surface models to test the facial surface superimposition areas. These models have been shown to have comparable accuracy to that of the 3D photos^16,45,46^. However, the area on the upper half of the forehead could not be tested since it was not included in the CBCT images. Another limitation is that the sample size was moderate and included individuals with different craniofacial patterns. Although this could have influenced the outcomes, there are limited effects expected at the facial areas used here as references for superimposition.

## Conclusion

The present study tested the performance of different 3D facial surface superimposition techniques in growing individuals and compared their outcomes to that of a standard technique, namely a voxel-based superimposition on anterior cranial base structures. A small rectangular area on the forehead plus an area encompassing the middle part of the nose and the lower wall of the orbital foramen provided comparable results to the standard technique and showed adequate reproducibility. Other reference areas that have been used so far were less reliable.

The utilization of the suggested method has potential to greatly expand the possibilities of this highly informative, risk free, and easily obtained 3D tool for the assessment of facial changes in growing individuals. The results of this study need to be confirmed on larger samples with different time spans between serial images and in other age groups.

## Supplementary Information

Supplementary Information.

## Data Availability

All outcome data are available as summary measures or representative images in the main text or the extended data. The surface models, protocols, and raw datasets generated and/or analyzed during the current study are available from the corresponding author on reasonable request.
