# Supplementary material for: Valid 3D surface superimposition references to assess facial changes during growth
Source: Sci Rep. 2021 Aug 12;11:16456. doi: 10.1038/s41598-021-95942-3 (PMC8361153; doi:10.1038/s41598-021-95942-3)
Supplement: Supplementary file 1 — Supplementary Information. [file 41598_2021_95942_MOESM1_ESM.docx]

**Valid 3D surface superimposition references to assess facial changes during growth**

Simeon T. Häner, Georgios Kanavakis, François Matthey, and Nikolaos Gkantidis

**Supplementary Figures:**


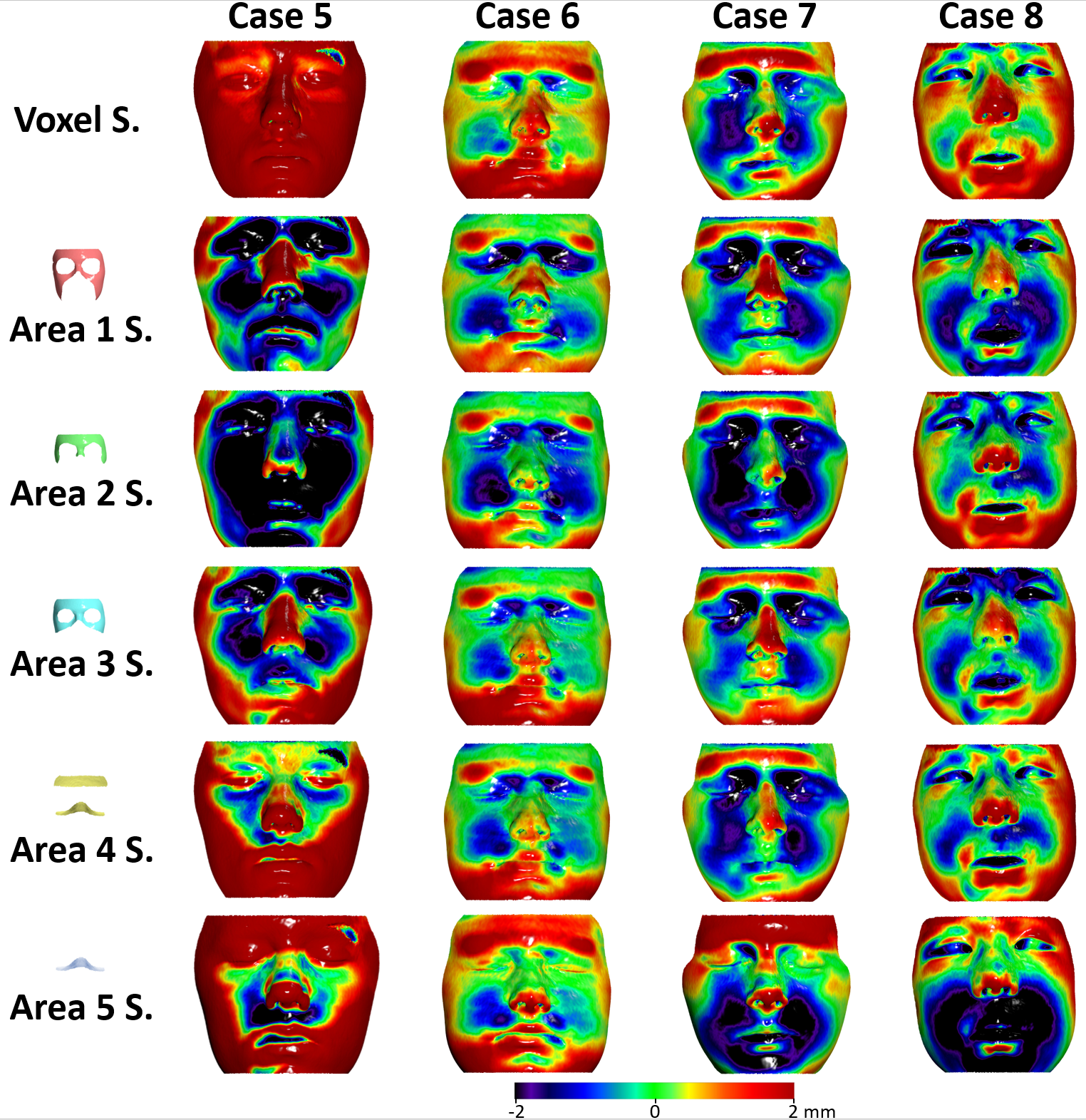


**Supplementary Figure 1.** Colour coded distance maps showing T0-T1 facial surface changes of four cases, as detected by anterior cranial base voxel-based or five different facial surface-based superimpositions. The T0 facial surface model was used as a reference. S.: Superimposition. All images were generated using Viewbox 4 software (version 4.1.0.1 BETA, http://www.dhal.com/viewboxindex.htm).


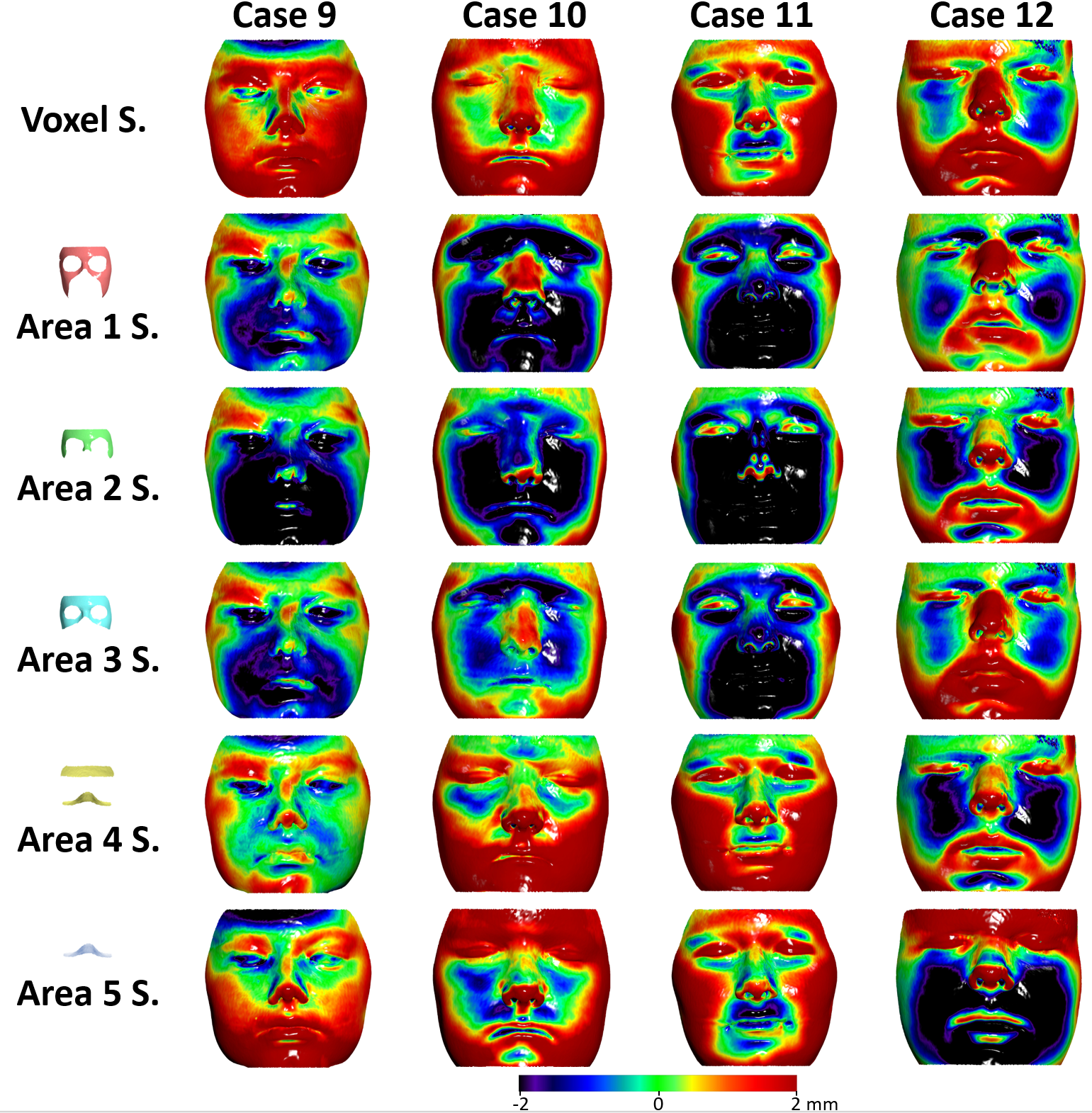


**Supplementary Figure 2.** Colour coded distance maps showing T0-T1 facial surface changes of four cases, as detected by anterior cranial base voxel-based or five different facial surface-based superimpositions. The T0 facial surface model was used as a reference. S.: Superimposition. All images were generated using Viewbox 4 software (version 4.1.0.1 BETA, http://www.dhal.com/viewboxindex.htm).


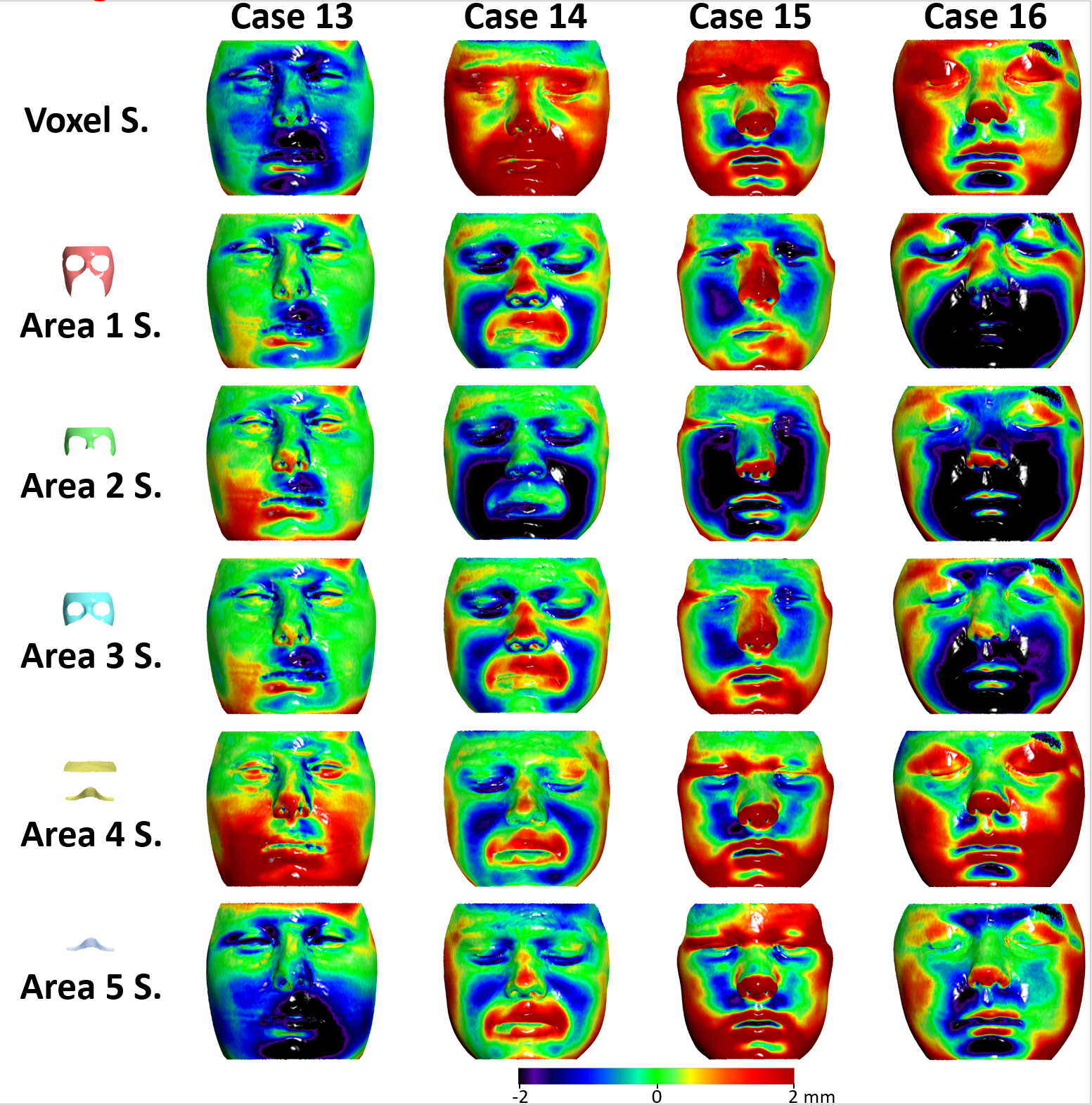


**Supplementary Figure 3.** Colour coded distance maps showing T0-T1 facial surface changes of four cases, as detected by anterior cranial base voxel-based or five different facial surface-based superimpositions. The T0 facial surface model was used as a reference. S.: Superimposition. All images were generated using Viewbox 4 software (version 4.1.0.1 BETA, http://www.dhal.com/viewboxindex.htm).


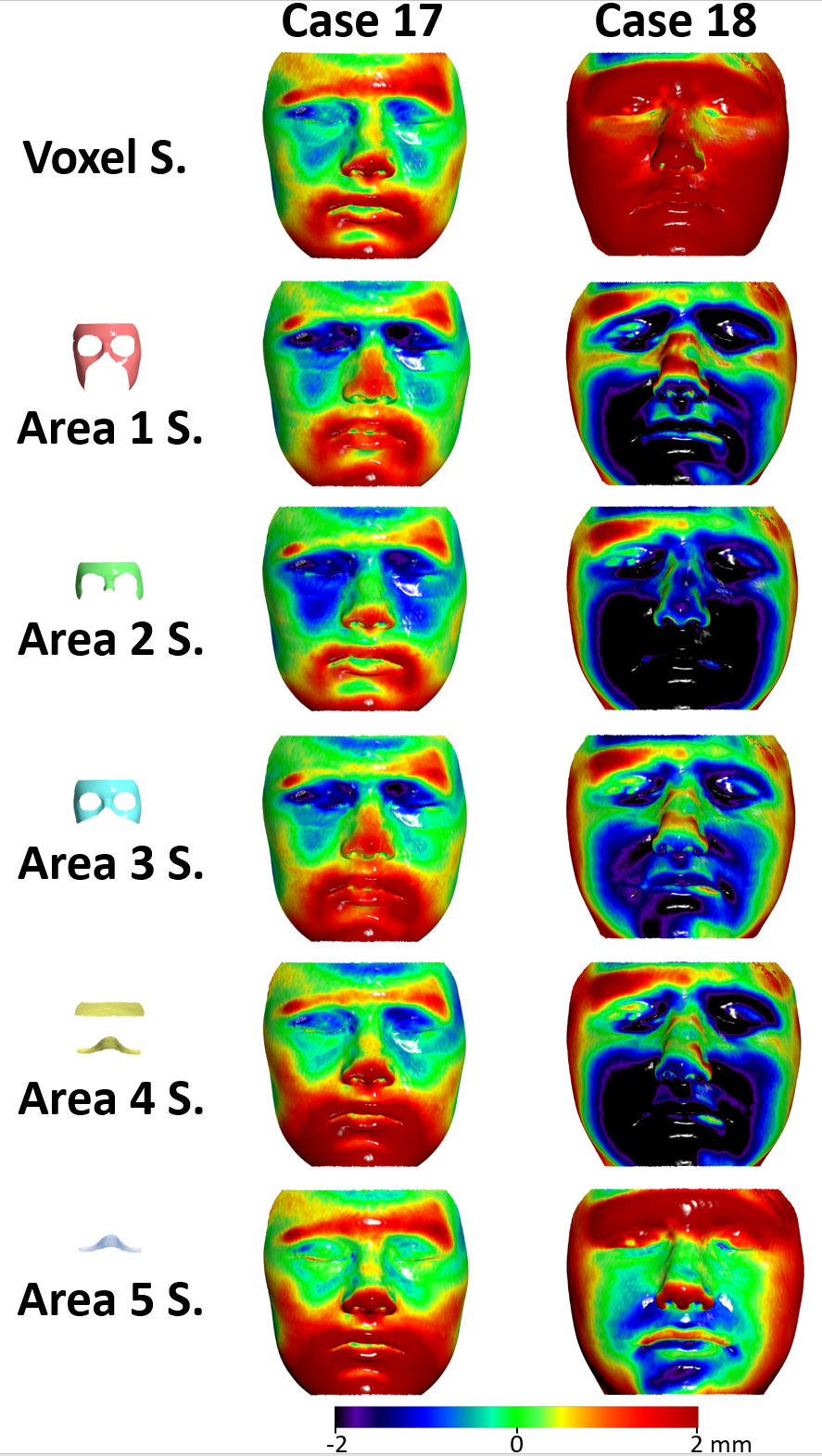


**Supplementary Figure 4.** Colour coded distance maps showing T0-T1 facial surface changes of two cases, as detected by anterior cranial base voxel-based or five different facial surface-based superimpositions. The T0 facial surface model was used as a reference. S.: Superimposition. All images were generated using Viewbox 4 software (version 4.1.0.1 BETA, http://www.dhal.com/viewboxindex.htm).


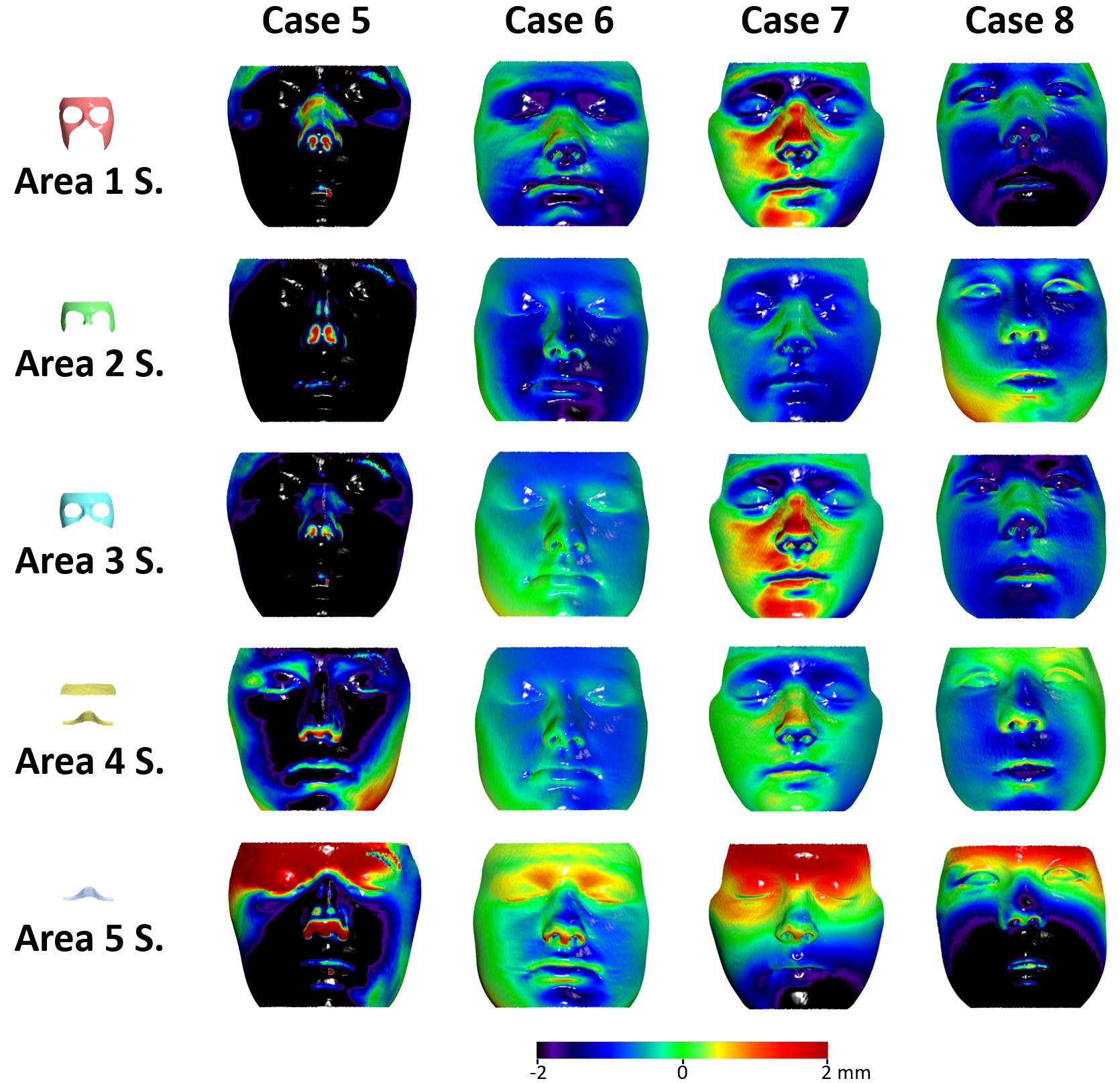


**Supplementary Figure 5.** Colour coded distance maps showing the differences in the outcomes (T1 models) of the five different facial surface-based superimpositions from the anterior cranial base voxel-based superimposition, on four cases. The voxel-based superimposition T1 surface model was used as a reference. S.: Superimposition. All images were generated using Viewbox 4 software (version 4.1.0.1 BETA, http://www.dhal.com/viewboxindex.htm).


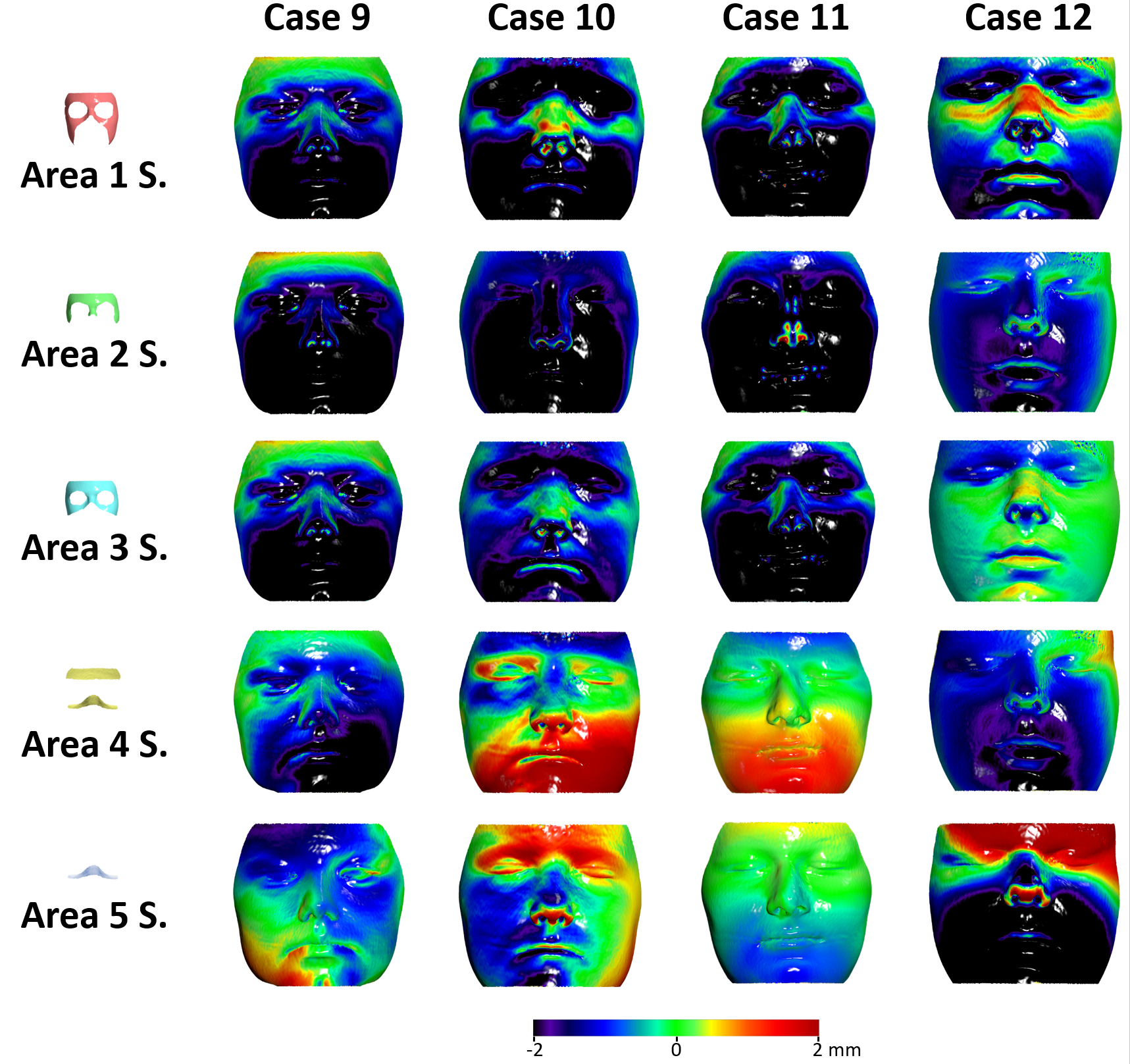


**Supplementary Figure 6.** Colour coded distance maps showing the differences in the outcomes (T1 models) of the five different facial surface-based superimpositions from the anterior cranial base voxel-based superimposition, on four cases. The voxel-based superimposition T1 surface model was used as a reference. S.: Superimposition. All images were generated using Viewbox 4 software (version 4.1.0.1 BETA, http://www.dhal.com/viewboxindex.htm).


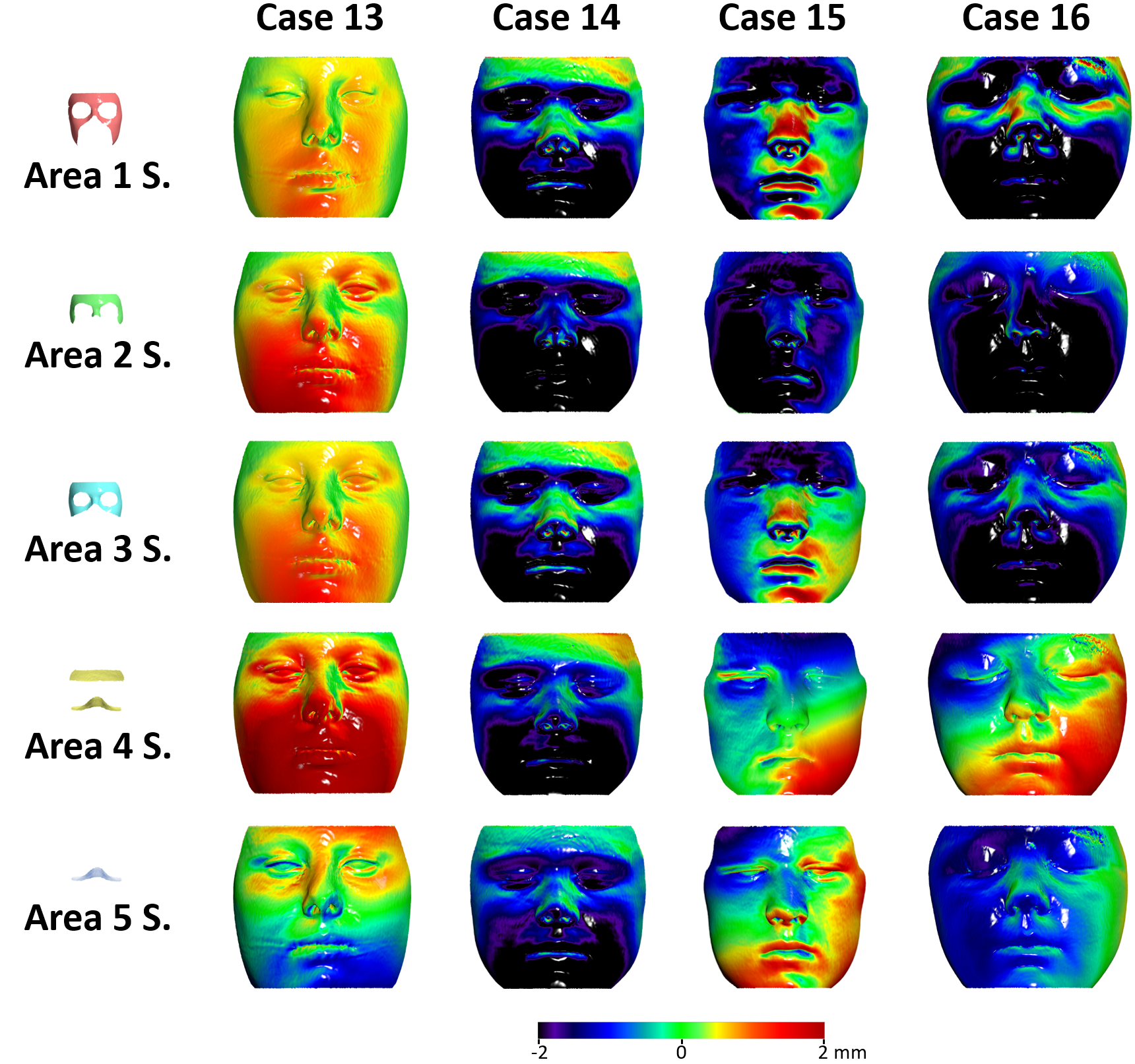


**Supplementary Figure 7.** Colour coded distance maps showing the differences in the outcomes (T1 models) of the five different facial surface-based superimpositions from the anterior cranial base voxel-based superimposition, on four cases. The voxel-based superimposition T1 surface model was used as a reference. S.: Superimposition. All images were generated using Viewbox 4 software (version 4.1.0.1 BETA, http://www.dhal.com/viewboxindex.htm).


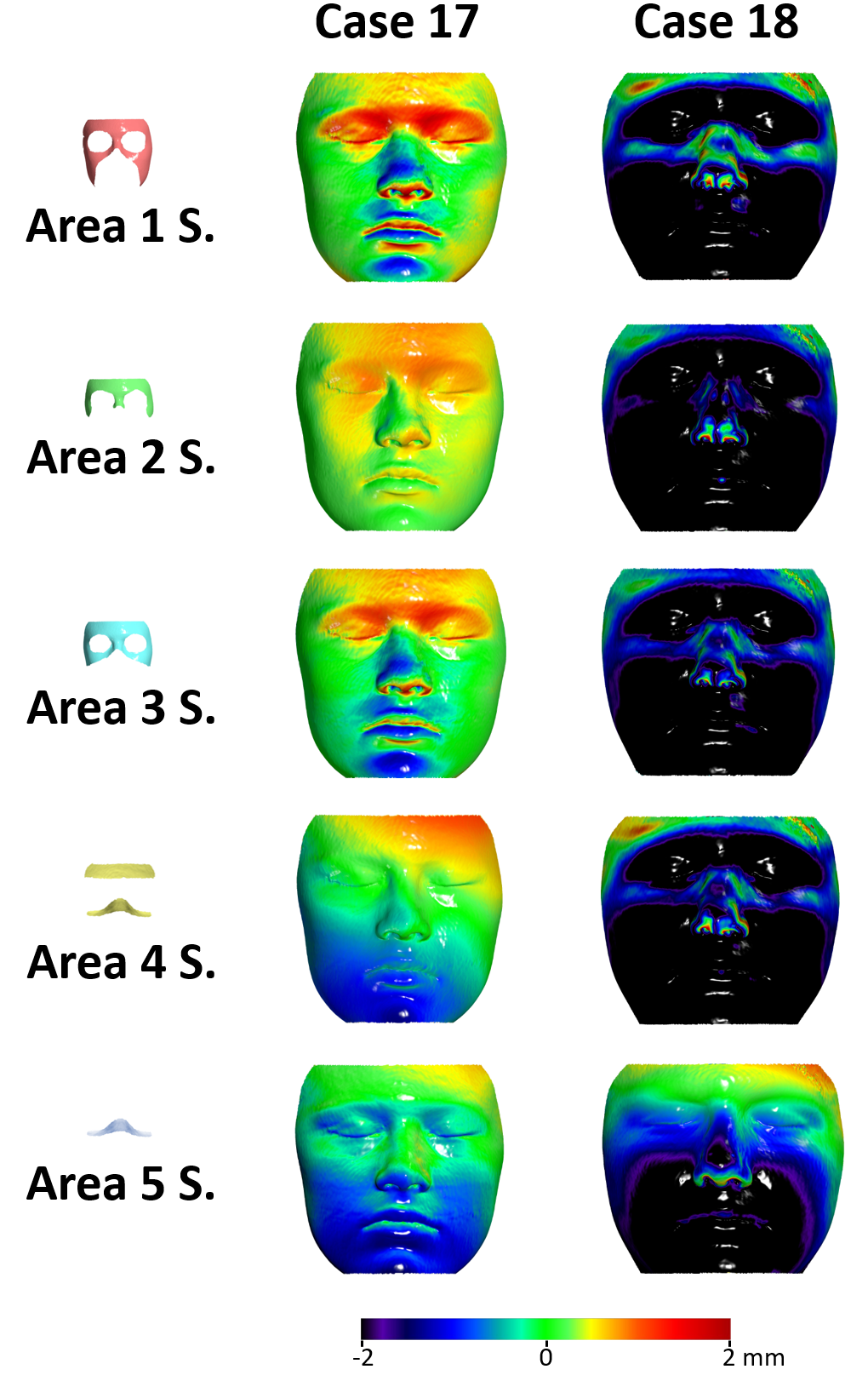


**Supplementary Figure 8.** Colour coded distance maps showing the differences in the outcomes (T1 models) of the five different facial surface-based superimpositions from the anterior cranial base voxel-based superimposition, on two cases. The voxel-based superimposition T1 surface model was used as a reference. S.: Superimposition. All images were generated using Viewbox 4 software (version 4.1.0.1 BETA, http://www.dhal.com/viewboxindex.htm).
